# Sensory Symptom Profiles and Co-Morbidities in Painful Radiculopathy

**DOI:** 10.1371/journal.pone.0018018

**Published:** 2011-05-09

**Authors:** Friederike Mahn, Philipp Hüllemann, Ulrich Gockel, Mathias Brosz, Rainer Freynhagen, Thomas R. Tölle, Ralf Baron

**Affiliations:** 1 Sektion Neurologische Schmerzforschung und -therapie, Klinik für Neurologie, Universitätsklinikum Schleswig-Holstein, Campus Kiel, Kiel, Germany; 2 CASQUAR GmbH, Computerassoziierte Qualitätssicherung und Rehabilitationsförderung, Bochum, Germany; 3 StatConsult GmbH, Magdeburg, Germany; 4 Zentrum für Anästhesiologie, Intensivmedizin, Schmerztherapie und Palliativmedizin, Benedictus Krankenhaus Tutzing, Tutzing, Germany; 5 Klinik für Neurologie, Technische Universität München, München, Germany; Charité-Universitätsmedizin Berlin, Germany

## Abstract

Painful radiculopathies (RAD) and classical neuropathic pain syndromes (painful diabetic polyneuropathy, postherpetic neuralgia) show differences how the patients express their sensory perceptions. Furthermore, several clinical trials with neuropathic pain medications failed in painful radiculopathy. Epidemiological and clinical data of 2094 patients with painful radiculopathy were collected within a cross sectional survey (pain*DETECT*) to describe demographic data and co-morbidities and to detect characteristic sensory abnormalities in patients with RAD and compare them with other neuropathic pain syndromes. Common co-morbidities in neuropathic pain (depression, sleep disturbance, anxiety) do not differ considerably between the three conditions. Compared to other neuropathic pain syndromes touch-evoked allodynia and thermal hyperalgesia are relatively uncommon in RAD. One distinct sensory symptom pattern (sensory profile), i.e., severe painful attacks and pressure induced pain in combination with mild spontaneous pain, mild mechanical allodynia and thermal hyperalgesia, was found to be characteristic for RAD. Despite similarities in sensory symptoms there are two important differences between RAD and other neuropathic pain disorders: (1) The paucity of mechanical allodynia and thermal hyperalgesia might be explained by the fact that the site of the nerve lesion in RAD is often located proximal to the dorsal root ganglion. (2) The distinct sensory profile found in RAD might be explained by compression-induced ectopic discharges from a dorsal root and not necessarily by nerve damage. These differences in pathogenesis might explain why medications effective in DPN and PHN failed to demonstrate efficacy in RAD.

## Introduction

Pain associated with chronic radiculopathy (RAD) is caused by compression or lesion of a dorsal root or its ganglion. Since the pain involves pathology of a peripheral nerve trunk it is thought to be mainly of neuropathic pain origin. In fact, the recently proposed new definition of peripheral neuropathic pain, i.e. *pain arising as a direct consequence of a lesion or disease affecting the peripheral somatosensory system* includes painful radiculopathy [1]. There are, however, differences in painful radiculopathies and the classical neuropathic pain syndromes, i.e. painful diabetic polyneuropathy (DPN) or postherpetic neuralgia (PHN), how the patients express their abnormal sensory perceptions. Furthermore, several recent clinical trials with medications which are effective in polyneuropathy and postherpetic neuralgia have failed to demonstrate superiority over placebo in painful radiculopathy [2], [3], [4].

Thus, the question arises whether pain generating mechanisms in patients with painful radiculopathy are different from those with other neuropathic pain syndromes although all patients have a nerve injury in common. In a recent study in patients with PHN and DPN we described five distinct subgroups of patients in both entities who are characterized by a specific sensory profile, a typical constellation and combination of neuropathic symptoms. We hypothesized that distinct pain-generating mechanisms are related to the specific sensory profiles in each of the patient subgroups [5].

In the present investigation we used the same approach to define subgroups of patients according to sensory profiles in a cohort of 2094 patients with painful radiculopathy and compared these profiles with the classical neuropathic pain syndromes, PHN and DPN. We analysed epidemiological and clinical data of painful radiculopathy patients who were collected within a cross sectional cohort survey in Germany (pain*DETECT*) performed in collaboration with the German Research Network on Neuropathic Pain (DFNS).

The aims were (1) to describe demographic data and co-morbidities in painful radiculopathy and (2) to detect characteristic sensory abnormalities in patients with painful radiculopathy and compare them with other neuropathic pain syndromes.

## Methods

### 1. Study population und data collection

**Figure 1:**
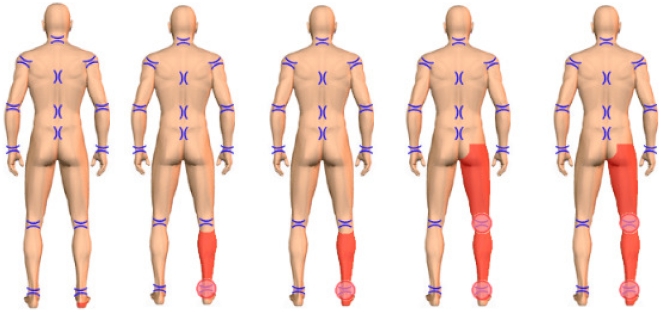
Pain distribution in patients with painful radiculopathy. Criteria for the selection of painful radiculopathy were the following: Leg pain had to be the predominant complaint whereas back pain was absent or of minor intensity. In order to select patients with neuropathic painful radiculopathy with the highest probability only patients who marked their most prominent pain (in red) in the following areas were included: (Foot) OR (foot AND shank) OR (foot AND shank AND thigh).

The study was performed at 450 outpatient centers throughout Germany, including general practitioners, rheumatologists, orthopaedists and pain specialists. In total 2094 patients presenting with neuropathic pain (painful radiculopathy, postherpetic neuralgia, painful diabetic neuropathy), at least 18 years old, were given hand-held computers (personal digital assistants, PDAs; Palm Tungsten E operating on the platform OS 5.4). They were requested to complete electronically questionnaires for the epidemiological and clinical survey and to mark their painful areas on a body drawing (see figure 1). In addition to standard demographic questions the following validated questionnaires were used to assess co-morbidities and sensory abnormalities: for sleep disturbances the Medical Outcomes Study sleep scale (MOS; [6]), for depressive disorders and panic and anxiety disorders the German-language Patient Health Questionnaire (PHQ-9, short form; [7]) and for sensory symptoms the pain*DETECT* questionnaire (PD-Q; [8]). Further examinations, e.g. neurophysiological studies, were not part of this large cross-sectional analysis.

The method of data acquisition was validated in an earlier study [9]. At intervals, PDAs were collected and data transfer to a central data base and data processing were performed under secure conditions, with anonymisation and encryption. Physicians did not receive a financial incentive for taking part in the epidemiological study. The study protocol was approved by the ethical committee of the University of Düsseldorf, and all participating patients gave written informed consent according to the Declaration of Helsinki.

### 2. Identification of painful radiculopathy and assessment of sensory symptoms

Patients with back pain may suffer from a variety of different pain syndromes which are mechanistically distinct [10]. First, nociceptive back pain is evoked by noxious stimulation of structures in the lumbar spine and is characterized by a dull and aching quality localized in the back. Second, somatic referred pain spreads into the lower limbs, most frequently the proximal areas and is of dull, aching and gnawing quality and is often difficult to localize. Somatic referred pain does not involve compression of nerve roots but is rather explained by a convergent afferent input on central neurons. Third, painful radiculopathy is induced by pathology of nerve root or its ganglion and is perceived along the length of the lower limb most frequently in the L5/S1 dermatomal distribution. The latter pain type is thought to be of neuropathic origin. From these clinical descriptions it is evident that in some cases it might be difficult to clinically distinguish between somatic referred pain and painful radiculopathy.

In order to circumvent this problem and minimize overlap the following approach was used to detect patients with painful radiculopathy with the highest level of security:

Only patients were included in the study in whom the leg pain was the predominant complaint whereas back pain was absent or of minor intensity. This selection was done based on pain body drawings performed by the patients on the palm top. The palm top device was equipped with a body drawing with 34 predefined body areas (Fig. 1). All patients marked (1) their body areas with the most prominent pain which was coded in red color and (2) their body areas in which the pain was radiating which was marked in green color. In order to select patients with neuropathic painful radiculopathy with the highest probability only patients who marked their most prominent pain in the following areas were included:

(Foot) OR (foot AND shank) OR (foot AND shank AND thigh).

To assess the sensory symptoms the pain*DETECT* questionnaire (PD-Q; [8]) was used which was also provided in the palm top device. The patients were asked to describe the abnormal sensory symptoms which they perceived in the body areas of their most intense pain (red areas). The patients rated the perceived severity of each symptom from 0–5 (never, hardly noticed, slightly, moderately, strongly, very strongly). In detail the questions address the following sensory symptoms: question 1 - spontaneous burning pain, question 2 - spontaneous prickling sensations, question 3 - pain evoked by light touch (allodynia), question 4 - spontaneous pain attacks, question 5 - pain evoked by thermal stimuli, question 6 - numbness, question 7 - pain evoked by slight pressure.

### 3. Statistics

Descriptive statistical analyses were performed with the SAS package, version 9.2. Data for graphics were transferred to MS Excel 2003. Relations between two dichotomous variables were assessed by 2×2 contingency tables, relations between categorial data in general using k×m contingency tables. Analysis of variances (Proc GLM) was used to evaluate differences in continuous variables between three groups of patients. Continuous variables were presented within tables by mean plus/minus standard deviation. Categorial data were tabulated using frequencies and percentages.

In order to identify relevant subgroups of patients who are characterized by a typical constellation of sensory symptoms a hierarchical cluster analysis was performed. To eliminate inter-individual differences of the general perception of sensory stimuli (differences in individual pain perception thresholds) the intensity scores of the questions were re-calculated for the cluster analysis. In detail, the given 0–5 score of each question was subtracted by the mean of all values marked in the 7 questions. In this individual score values above 0 indicate a sensation which is more intensive than the individual mean pain perception, values below 0 indicate a sensation which is less intensive than the individual mean pain perception.

As there are no special a-priori-assumptions about the distance measures and the number of clusters for this heuristic approach, we used the commonly recommended hierarchical WARD-approach with a squared Euclidian distance measure. As there are no objective and compelled rules for determination of an optimal cluster number we used 3 criteria: the development of values of the WARD fusion algorithm with respect to cluster numbers, practical decisions about minimal group numbers and decisions about sense of combining groups as regards content.

Using these criteria we identified a 5-cluster solution to be the optimal compromise. We applied the 5-cluster solution in the radiculopathy cohort and in the cohort of DPN/PHN patients separately. To prove the evidence of the solution a k-means cluster analysis which rearranges cases for better fitting was performed. The clusters are represented by the patterns of the questionnaire scores, thus showing the typical pathological structure of the respective group. As this is a heuristic approach no statistical analysis was performed.

## Results

### 1. Demographic data and co-morbidities in painful radiculopathy and other neuropathic pain syndromes

A total of 2094 patients with painful radiculopathy took part in the survey and were compared with 2121 patients with other neuropathic pain syndromes (1623 DPN and 498 PHN patients). The latter data have been published elsewhere [5]. The demographic profile and the severity of co-morbidities of the radiculopathy patients are shown in Table 1. Compared to the group of patients suffering from DPN, patients with RAD were slightly more depressive (PHQ-9-score moderate (10–19) 37.2% vs. 31.6%, p<0.001). Anxiety disorders and somnolence, however, occurred a little less frequently in patients with RAD than in patients with DPN (anxiety disorders: 4.6% vs. 8.6%, p<0.001; somnolence 39.8±21.8 vs. 46.5± ´′22.4, p<0.001, compare [5]).

**Table 1:** Demographic and clinical characteristics of patients with painful radiculopathy (RAD).

Entity	Painful radiculopathy
Patients (*n*, %)	2094 (100.0)
Male (*n*, %)	872 (41.6)
Female (*n*, %)	1222 (58.4)
Age (years)* total	59.4±14.4/50; 70
Height (cm)** males	177.0±8.2
females	164.5±7.0
Weight (kg)** males	88.2±16.0
females	75.3±16.4
BMI (kg/m^2^)** males	28.2±5.0
females	27.9±6.7
**PHQ-9 score, depression**	
None (0–4)	22.8%
Mild (5–9)	35.2%
Moderate (10–19)	37.2%
Severe (20–27)	4.8%
Panic/anxiety disorder present	4.6%
**MOS sleep scale**	
Sleep disturbances [0;100]	44.5±25.2
Optimal sleep	37.1%
Somnolence [0;100]	39.8±21.8
Sleep quantity (hours)	6.1±1.6
Sleep adequacy [0;100]	51.3±28.0

*mean ± standard deviation. BMI, body mass index; P25/P75, 25% and 75% percentiles;

n.s., not significant.

**mean ± standard deviation.

### 2. Sensory symptoms in painful radiculopathy and other neuropathic pain syndromes

The VAS intensity values for “worst pain”, “average pain” and “current pain” were similar in RAD (7.4±2.1; 5.8±2.1; 5.1±2.6) and PHN (7.4±2.1; 5.5±2.0; 5.0±2.4) and slightly less in DPN patients (6.4±2.6; 5.0±2.3; 4.6±2.5; p<0.001 for all comparisons). The seven questions of the pain*DETECT* questionnaire address the quality and intensity of specific sensory symptoms (Tab. 2). The patients could rate the perceived severity of each of these symptoms from 0–5 (never, hardly noticed, slightly, moderately, strongly, very strongly). In Table 2 the frequency of the sensory disturbances that were regarded as clinically relevant (i.e. if the patients marked a score of >3, strongly, very strongly) is shown for each question.

**Table 2:** Pain and sensory symptoms in patients with painful radiculopathy (RAD).

Entity	Painful radiculopathyN = 2094
VAS worst pain*	7.4±2.1
VAS average pain*	5.8±2.1
VAS current pain*	5.1±2.6
Clinical relevant complaint (score >3)**	
Q1, burning	25%
Q2, prickling	26%
Q3, allodynia	10%
Q4, attacks	32%
Q5, thermal	8%
Q6, numbness	16%
Q7, pressure	21%

*mean ± standard deviation. Score >3, strongly, very strongly.

Burning pain occurred least frequent in RAD (25%) followed by DPN and PHN (33%, 54%; p<0.001). Prickling sensations also occurred least frequent in RAD (26%) compared to DPN and PHN (35%, 38%; p<0.001). Importantly, clinically relevant touch evoked allodynia and thermal induced pain were very uncommon symptoms in RAD (10%, 8%), followed by DPN (18%, 14%) and PHN (47%, 31%; p<0.001). Severe pain attacks were described similarly frequent in RAD and DPN and nearly in half of the PHN patients (32%, 29%, vs. 46%; p<0.001). Numbness was a prominent descriptor in DPN (30%) and evenly distributed in RAD and PHN (16%, 14%; p<0.001). Pain induced by slight pressure occurred in RAD patients nearly as often as in DPN patients and half as often as in PHN patients (21% vs. 22% and 42%; p<0.001). For detailed results of DPN and PHN analysis please see [5].

**Figure 2:**
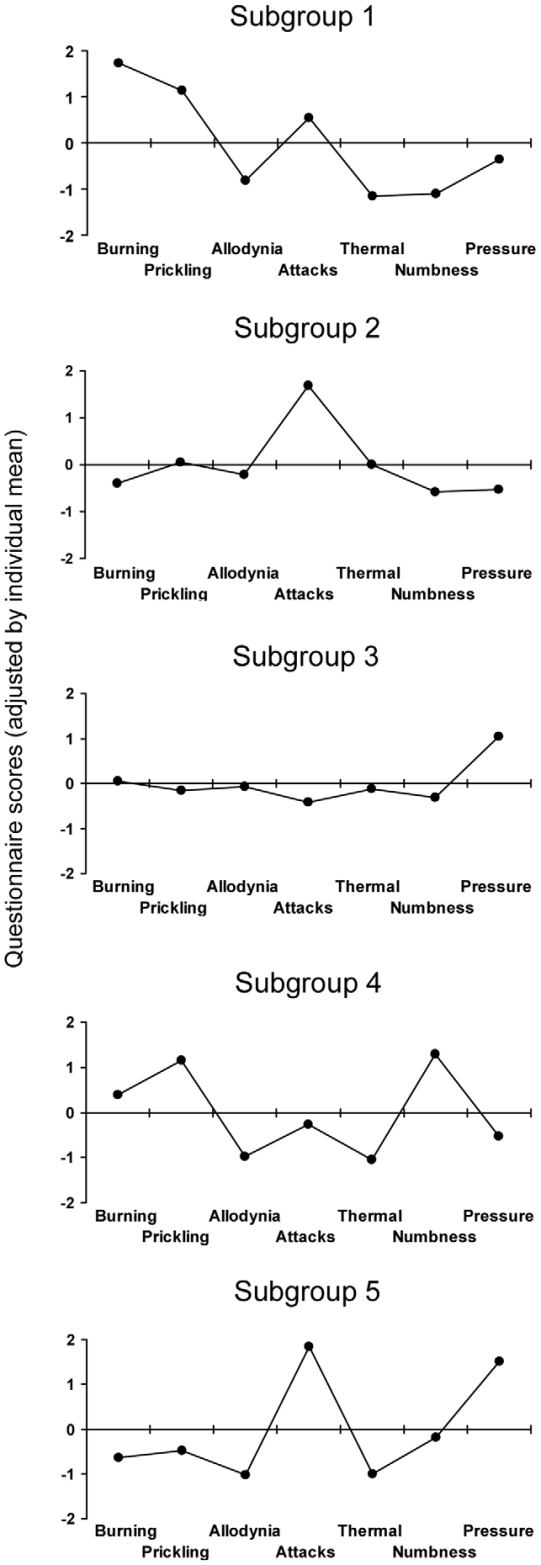
Subgroups of patients based on sensory symptoms. To identify relevant subgroups of patients who are characterized by a characteristic symptom constellation a hierarchical cluster analysis was performed. The clusters are represented by the patterns of questionnaire scores (adjusted individual mean), thus showing the typical pathological structure of the respecting group. By using this approach five clusters with distinct symptom profiles could be detected in the RAD cohort. Sensory profiles show remarkable differences in the expression of the symptoms. Subgroup 5 was unique for patients with painful radiculopathy. RAD = painful radiculopathy.

In addition to the frequencies of each of the sensory symptoms the patients also showed typical combination patterns of symptoms, i.e. typical sensory profiles. A cluster analysis was performed to identify relevant subgroups of patients who present with a characteristic constellation of sensory neuropathic symptoms and to detect these profiles in RAD. Table 3 and Figure 2 show the different clusters with distinct symptom profiles. In the 5 cluster-solution we found sensory profiles with remarkable differences in the expression of the experienced symptoms. When compared to DPN and PHN, four subgroups were present in all three different entities with some differences in relative frequency. The sensory profiles of DPN and PHN are shown in (5). Subgroup 1 was found in 18% of RAD patients, in 13% of DPN patients and in 34% of PHN patients (p<0.001). Subgroup 2 was shown in 16% both in RAD and DPN patients and in 11% of PHN patients. In 29% of RAD patients, 37% of DPN patients and 25% of PHN patients subgroup 3 was found (p<0.001). Subgroup 4 occurred in 22% and 26% of RAD and DPN patients, but just in 5% of PHN patients (p<0.001). Interestingly, the subgroup 5 could only be detected in patients with painful radiculopathy.

**Table 3:** Distribution of sensory symptom profiles (clusters) in patients with painful radiculopathy (RAD).

Subgroups(sensory profiles)	RAD[%]
Subgroup 1	18
Subgroup 2	16
Subgroup 3	29
Subgroup 4	22
Subgroup 5	15

Subgroup 1 to 4 occurred also in patients with DPN and PHN (see [5]). Subgroup 5 was unique for patients with painful radiculopathy. Numbers represent frequencies in percent.

## Discussion

The aim of the present investigation was to compare three etiologically different neuropathic pain syndromes, i.e. painful radiculopathy (RAD), painful diabetic neuropathy (DPN) and postherpetic neuralgia (PHN) and to describe similarities and differences in demographic data, co-morbidities and sensory perceptions. The study revealed three main findings:

1. The frequency and severity of common co-morbidities in neuropathic pain, i.e., depression, sleep disturbance and anxiety, are similar across conditions.
2. Compared to other peripheral neuropathic pain syndromes touch-evoked allodynia and thermal hyperalgesia are relatively uncommon in painful radiculopathy (only about 10%).
3. One distinct sensory symptom pattern (sensory profile), a combination of severe painful attacks and pressure induced pain with the lack of spontaneous pain, allodynia and thermal hyperalgesia, was found to be characteristic for patients with painful radiculopathy.

### 1. Demographic data and co-morbidities in painful radiculopathy and other neuropathic pain syndromes

The literature indicates a clear link between psychological variables and back pain [11], [12]. The prevalence of major depression in patients with chronic low back pain is approximately three to four times greater than that reported in the general population [13]. Furthermore, psychological factors (notably distress, depressive mood, and somatisation) are implicated in the transition to chronic low back pain [14]. A direct comparison of psychological variables in painful radiculopathy, DPN and PHN has never been performed. Interestingly, the results of the present study revealed that the incidence of psychological co-morbidities associated with painful radiculopathy did not differ considerably from that in other neuropathic pain disorders. Similar findings were described when comparing psychological functions in patients with nociceptive low back pain and postherpetic neuralgia [15]. Thus, differences in the treatment response between painful radiculopathy and other neuropathic pain syndromes are unlikely related to differences in the incidence of co-morbidities.

### 2. Sensory symptoms in painful radiculopathy and other neuropathic pain syndromes

Sensory disturbances were considered as clinically relevant if the patients replied to the questions with a score of >3 (strongly, very strongly) (Tab. 2). Although patients of all three entities chose very similar descriptors to characterize their sensory perceptions the frequency of symptoms differed somewhat across the different entities. Pain attacks and pressure induced pain occurred relatively frequently in RAD (in a similar frequency as in DPN). Numbness which indicates a loss of sensory innervation occurred in 15% of patients with painful radiculopathy, but was much more pronounced in DPN patients (30%). The important difference found in this study is that touch-evoked allodynia and thermal hyperalgesia are relatively uncommon in painful radiculopathy patients (only about 10%). This paucity of evoked sensory symptoms in RAD is somewhat unexpected. A potential explanation might be that the anatomical site of the nerve lesion differs between the syndromes. In DPN and PHN the peripheral branches of the primary afferent neurons or the dorsal root ganglion itself are affected by the disease process. Animal experiments have shown that partial lesions of the peripheral nerve branches lead to Wallerian degeneration of peripheral axons and induce a hyperexcitable state of the remaining neurons by up-regulation of a variety of novel channels and receptors [16], [17], [18]. In contrast, in cases of painful radiculopathy the compression and lesion is located at the nerve root, i.e. proximally to the dorsal root ganglion. This site of damage leads to degeneration of the central branches of the afferent neurons which terminate in the spinal cord dorsal horn and leaves the peripheral branches of the neuron intact.

To demonstrate differences between the two lesion sites an animal model of radiculopathy was directly compared to a model of peripheral nerve lesion. Despite the fact that pinprick allodynia, a sensory phenomenon which was not part of the present survey, was similar in both animal models, significant differences in spinal cord gene expression could be depicted [19]. In fact, there was only little overlap between genes altered in each model, suggesting that the site of injury produces distinct pathophysiological mechanisms. The authors speculated that “these distinct mechanisms in neuropathic versus radicular pain may implicate unique drug therapies for these types of chronic pain syndromes”.

The different lesion sites also lead to specific distributions of sensory symptoms. In radiculopathy and radiculoneuropathy the distribution is dermatomeric and non length-dependent whereas polyneuropathies show a length-dependent distribution.

Modern concepts hypothesize that sensory abnormalities and in particular the individual pattern of sensory symptoms might allow insight into the underlying pathophysiological mechanisms of pain generation. In light of this hypothesis we performed a cluster analysis to identify relevant subgroups of patients who demonstrate characteristic sensory profiles (Tab. 3, Fig. 2). This analysis revealed four subgroups of patients with characteristic sensory profiles which could be identified in all three conditions. The frequency, however, differed between the entities.

Subgroup 1 occurs nearly three times more frequently in PHN than in DPN and RAD (34%, 13%, 18%). The prominent features in this subgroup are moderate to strong spontaneous burning pain in combination with slight to moderate dynamic mechanical allodynia. Numbness was nearly ever noticed in this subgroup which indicates a preserved innervation of the skin without any signs of degeneration.

The dominant symptom of subgroup 2 is severe and clinical relevant pain attacks. This symptom constellation occurs in 16% of RAD, in 16% of DPN and in 11% of PHN patients. These patients very likely experience the classical neuropathic shooting pain which occurs spontaneously for seconds, comparable to the attacks in trigeminal neuralgia.

Patients who have been classified into subgroup 4 suffer from considerable burning pain and paresthesias but do not have relevant mechanical allodynia, thermal hyperalgesia and pain attacks. In contrast, numbness is a very prominent symptom. This symptom constellation indicates a severe deafferentation of the affected skin. Patients with painful radiculopathy and DPN show this symptom pattern much more frequently than PHN patients (22%, 26%, 5%). These findings are in line with results obtained in a group of patients with painful radiculopathy using quantitative sensory testing which revealed a selective loss of vibration detection, detection of v. Frey hair contact, and cold detection in the affected dermatomes. Allodynia and hyperalgesia was rare [20]. A length-dependent denervation of the skin nicely explains these findings.

One sensory profile, however, was found to be characteristic for patients with painful radiculopathy and does not occur in the other neuropathic pain conditions. This subgroup is characterized by a combination of severe painful attacks and pressure induced pain whereas spontaneous pain, allodynia and thermal hyperalgesia are only mildly present. It could be found in 15% of patients with RAD. Obviously, painful symptoms in this group are fundamentally different from perceptions that are experienced by DPN and PHN patients. What makes this sensory perception so unique for painful radiculopathies?

Many patients with back and leg pain use the descriptor “pain attacks” if they want to express that even the slightest movement of the affected lumbar spine is capable of inducing a very severe, short lasting pain which ceases immediately after seconds. Very similar sensory phenomena could be evoked in patients who underwent surgery for disc herniation. Sutures were placed around the nerves during surgery and led out through the wound [21]. When the patient was awake the sutures were pulled and the patient described the sensory perceptions. The evoked sensation had a lancinating, shocking and electric quality and travelled along the length of the lower limb. Physiologically, it is thought that these attacks are evoked by compression-induced ectopic discharges emanating from a dorsal root or its ganglion which are activated by the slightest movement [22]. Disc herniation and inflammation of the affected nerve seems to be the critical pathophysiological process [23], [24]. Consequently, the associated sensory profile does not occur in other neuropathic pain syndromes and most likely reflects the clinical phenomenon which is termed “radicular pain”.

The question arises whether this subgroup can really be summarized under the definition of neuropathic pain. It is believed that radicular pain is a classical feature of most radiculopathies, but clearly it can also occur in the absence of a neuropathy of the root, i.e., in the absence of any nerve damage [25]. The paucity of spontaneous sensation, allodynia and thermal hyperalgesia in this patient group also argues against major peripheral nerve damage. If this is true, many patients who fall into this subgroup would to a large extend suffer from pain mechanisms which are different from other neuropathic pain states. Consequently, it would not be surprising if medications that are efficacious in DPN and PHN might fail in some of the patients with radicular pain.

These phenotypic differences are certainly not the only variables which might determine the response to analgesic treatments. This is also influenced by genetic susceptibility and psychological factors such as catastrophizing and expectation which were not assessed in the present investigation. However, it might be possible that differences in sensory phenotypes explain some of the variance in treatment response and might, thus, be one puzzle piece to establish a more personalized treatment approach in the future. It might be possible to use this information to select the optimal patients for treatment with neuropathic pain drugs by their individual sensory profiles.

### Conclusion

Despite many similarities in sensory symptoms there are two decisive differences between painful radiculopathy and other neuropathic disorders. (1) The site of the nerve lesion in radiculopathy is often located proximal of the dorsal root ganglion which might explain the paucity of mechanical allodynia and thermal hyperalgesia and distinct underlying pathophysiological mechanisms. (2) Patients with painful radiculopathy often describe an evoked unpleasant sensation of lancinating, shocking or electric quality whereas in painful polyneuropathies and postherpetic neuralgia spontaneous burning pain and allodynia dominate the clinical picture. The pathophysiology of pain generation in this subgroup of radiculopathy is likely to be different from other painful neuropathies and might be explained by compression-induced ectopic discharges from a dorsal root and not necessarily by nerve damage. These differences might in part explain the failure of medications which are effective in classical neuropathic pain syndromes.
